# Recyclable Enzymatic Hydrolysis with Metal–Organic Framework Stabilized Humicola insolens Cutinase (HiC) for Potential PET Upcycling

**DOI:** 10.1021/cbe.4c00101

**Published:** 2024-08-30

**Authors:** Audrianna Wu, Fanrui Sha, Shengyi Su, Omar K. Farha

**Affiliations:** † International Institute for Nanotechnology and Department of Chemistry, Northwestern University, 2145 Sheridan Road, Evanston, Illinois 60208, United States; ‡ Department of Chemical and Biological Engineering, Northwestern University, 2145 Sheridan Road, Evanston, Illinois 60208, United States

**Keywords:** Protein encapsulation, biocatalysis, metal−organic frameworks, polyethylene terephthalate recycling, chemical upcycling

## Abstract

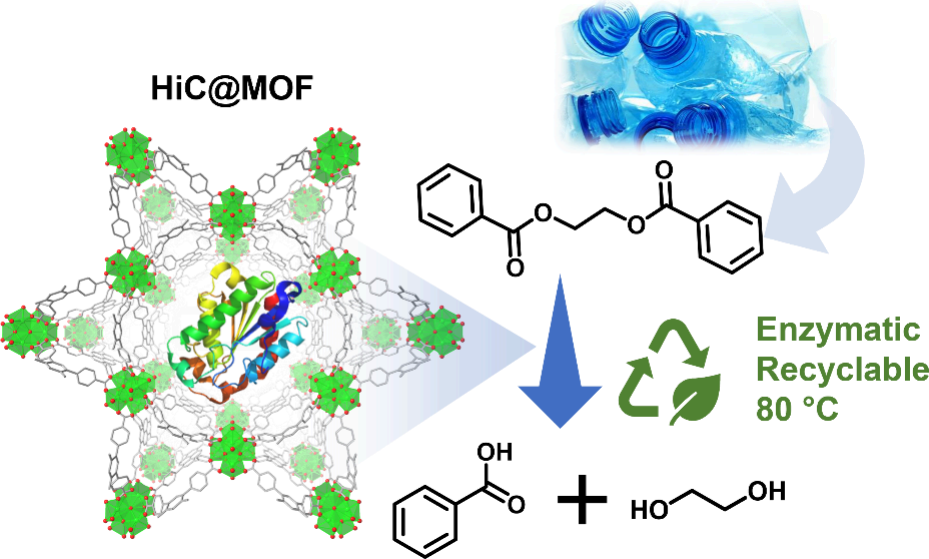

The degradation and recycling of plastics, such as poly(ethylene terephthalate) (PET), often require energy-intensive processes with significant waste generation. Moreover, prevalent methods primarily entail physical recycling, yielding subpar materials. In contrast, upcycling involves breaking down polymers into monomers, generating valuable chemicals and materials for alternative products. Enzyme-catalyzed depolymerization presents a promising approach to break down PET without the need for extreme conditions and unstable or toxic metal catalysts, which are typical of traditional recycling methods. However, the practical application of enzymes has been hindered by their high cost and low stability. In this study, we stabilized the enzyme Humicola insolens cutinase (HiC) by encapsulating it within a mesoporous zirconium-based metal–organic framework, NU-1000. HiC@NU-1000 exhibited a quantitative degradation of the PET surrogate, ethylene glycol dibenzoate (EGDB), with greater selectivity than native HiC in producing the fully hydrolyzed product benzoic acid in partial organic solvent. Additionally, the heterogeneous catalyst is also active toward the hydrolysis of PET and has demonstrated recyclability for at least four catalytic cycles. The HiC@NU-1000 model system represents a promising approach to stabilize industrially relevant enzymes under conditions involving elevated temperatures and organic solvents, offering a potential solution for relevant protein-related applications.

## Introduction

Plastic products have benefited our society with great convenience due to their low production costs and high stability. However, its extensive use leaves us with large amounts of highly stable waste. Recent reports show that over 200 million tons of plastic waste are generated annually worldwide, with less than 20% of plastic waste recycled properly.^1^ Leakage of plastic waste threatens the safety of food and water sources.^1,2^ Specifically, polyethylene terephthalate (PET) makes up over 40% of single-serve beverage packing in the United States, with over 90% of them ending up in landfills.^3^ With the accumulation of plastic waste, the pressure to find efficient, cost-effective, and green methods of recycling plastic also increases. However, most plastic recycling focuses on mechanical recycling and energy recovery through combustion, producing low-grade recycled products and additional pollutants.^4,5^ In contrast, chemical recycling, also known as polymer upcycling, depolymerizes polymer building blocks, such as the hydrolysis of PET into terephthalic acid (TPA) and ethylene glycol (EG).^6^ This method has great potential in producing high quality recyclable products with minimal waste.^7,8^ However, the high stability of these polymers makes their breakdown and chemical recycling challenging. Homogenous metal catalysts for these processes can be highly sensitive to water, oxygen, and metal salt contaminants and are often difficult to be separated from the final products.^9^ On the other hand, heterogeneous catalysis processes often require high temperature and pressure to soften the polymers and increase contact with the solid catalysts, which often leads to low selectivity and the formation of undesired byproducts.^8^ Therefore, selective, air-stable heterogeneous catalysts that operate at relatively low temperatures (150 °C or less) with facile separation from the products would be highly desirable for the application of polymer upcycling.

The use of enzymes as biological catalysts in polymer upcycling can avoid high temperatures and harsh chemical conditions typically required for upcycling.^6,10−12^ This environmentally friendly alternative approach to chemical upcycling is particularly promising due to the catalysts’ high efficiency and selectivity under mild reaction conditions. Humicola insolens Cutinase (also known as HiC or Novozym 51032) has been reported as an efficient PET depolymerization enzyme.^6,13,14^ However, similar to many other biocatalysts, HiC suffers from low solvent tolerance and limited thermal stability and is homogeneous, which makes catalyst separation and recovery difficult.^15^ At temperatures below the boiling point of PET, dissolving or swelling the polymers in organic solvent can maximize contact of the substrate with the catalysts. However, PET is only soluble in a few solvents, many of which denature proteins due to the disruption of hydrophobic effects essential for protein folding.^16^

Various techniques have been developed to stabilize enzymes against denaturants, such as heat, mechanical forces, various salt contents, and proteases. These techniques include but are not limited to immobilization through cross-linking, encapsulation through matrix entrapment or by micro- to nanosized capsules, PEGylation, and protein engineering.^17^ Among these methods, immobilization has been an effective technique in improving the stability and recyclability of enzymes with wide adaptability and minimal chemical modification required on the protein.^13,17,18^ To date, most reported immobilization materials are amorphous solids such as polymeric beads and porous silica,^19^ which can be hard to design, control, characterize, and systematically improve upon. Metal–organic frameworks (MOFs),^20^ on the other hand, can be designed and optimized as hosts for protein encapsulation due to their highly porous, crystalline, and modular nature^21^ and have demonstrated great potential as single-molecule traps for enzyme encapsulation.^22−24^ The geometry and chemical properties of the frameworks can be optimized toward the encapsulation and catalysis of specific protein,^22,24^ and the framework itself can also act as an anchor for additional catalytic reactions.^25^

**Figure 1:**
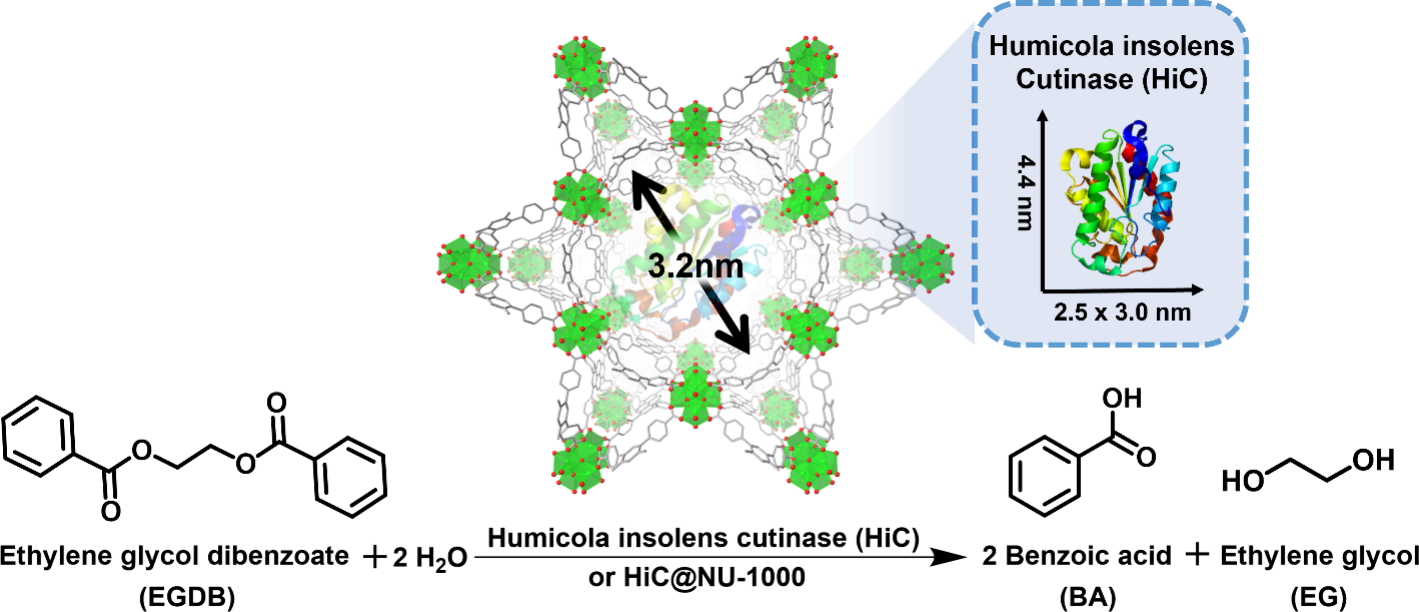
Schematic of HiC (Humicola insolens Cutinase) encapsulated in NU-1000 (HiC@NU-1000) and the hydrolysis of ethylene glycol dibenzoate (EGDB) into benzoic acid and ethylene glycol.

Humicola insolens Cutinase (HiC, Novozym 51032) is an enzyme which has shown great promise for PET hydrolysis with high selectivity.^6,14^ However, most existing systems are homogeneous, making product separation and HiC recovery challenging. To overcome this challenge, we chose to encapsulate HiC (2.5 × 3.0 × 4.4 Å^3^, PDB: 4OYY),^26^ with a highly chemically stable Zr-based MOF NU-1000, which contains mesopore sized 3.2 nm.^27,28^ Building upon our previous knowledge that enzymes can be stabilized against denaturing organic solvent and heterogenized for multiple rounds of catalysis via encapsulation in MOFs,^18^ we developed a robust, recyclable HiC-based biocatalyst (HiC@NU-1000) for the hydrolysis of ethylene glycol dibenzoate (EGDB) as a dimer representative for the larger PET molecule. Through systematic investigation, we optimized the encapsulation conditions for HiC, reaction conditions, and reaction solvent balanced between substrate solubility and enzyme activity (Figure 1). Through product quantification monitored by ^1^H NMR, we were able to demonstrate an active and recyclable HiC@NU-1000 with superior catalytic properties compared to the free HiC enzyme toward both EGDB and PET.

## Results and Discussion

### Developing HiC Encapsulation and EGDB Hydrolysis Conditions

**Figure 2:**
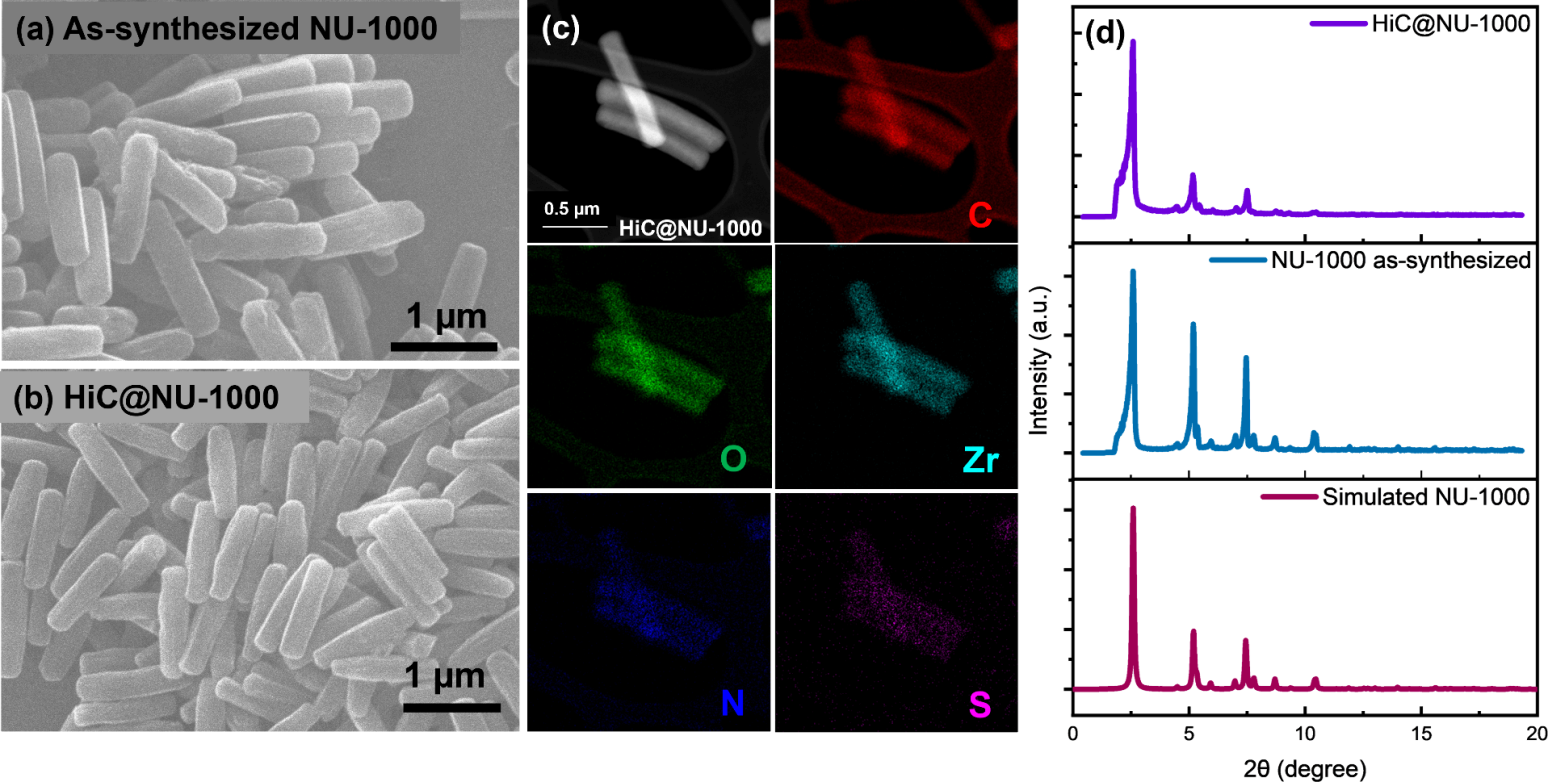
Physical characterization of NU-1000 and HiC@NU-1000, including (a,b) scanning electron microcopy (SEM) images, (c) HAADF-STEM image and STEM-EDS mapping signal showing spatial distribution of carbon, oxygen, zirconium, nitrogen, and sulfur elements in HiC@NU-1000, and (d) powder X-ray diffraction (PXRD) patterns of NU-1000 and HiC@NU-1000.

NU-1000 was synthesized based on a reported procedure (Figure S1).^27^ Encapsulation conditions such as salt concentration and pH of the solution have been shown to be highly influential to the kinetics of protein intraparticle diffusion and its encapsulation kinetics.^18,24,29^ Therefore, encapsulation of HiC in NU-1000 was first tested with Tris buffer between 0 and 1000 mM at pH 7.5 (Figure S2). The HiC encapsulation amount was determined by Bradford assay (Figure S3), and we found that the 500 mM condition appeared to yield the highest HiC loading while maintaining uniform enzyme distribution and the structural integrity of NU-1000 (Figure 2). We also observed that the addition of glycerol enhances the encapsulation of HiC to up to 10 wt % at 525 μg of HiC in 5 mg of NU-1000 (Figure S2b).

**Figure 3:**
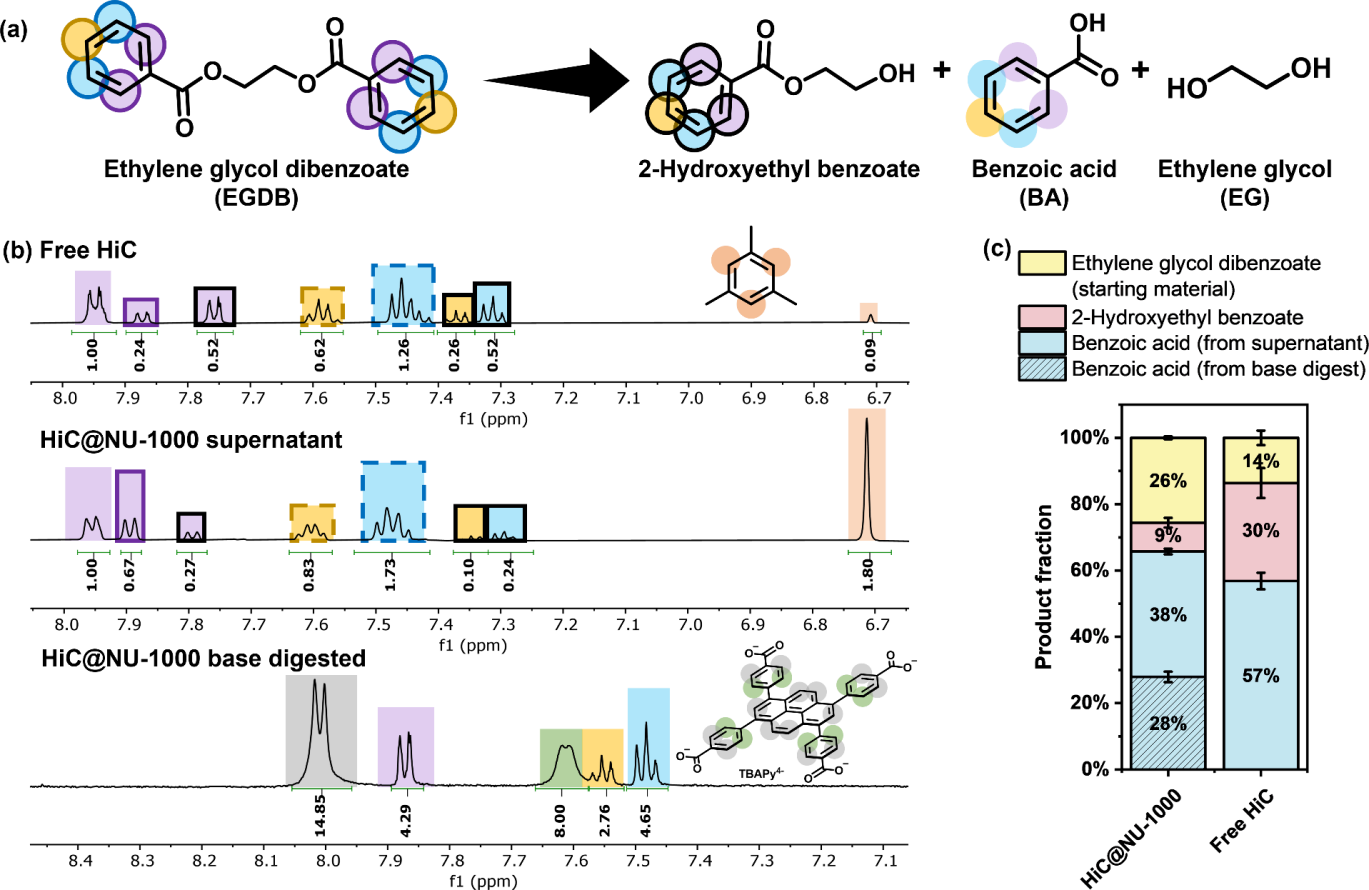
(a) Products from the hydrolysis of ethylene glycol dibenzoate (EGDB), which include 2-hydroxyethyl benzoate, benzoic acid, and ethylene glycol. (b) ^1^H NMR spectra of the reactions mixture after 48 h and the base-digested HiC@NU-1000 identifying the starting material EGDB, and its hydrolysis products 2-hydroxyethyl benzoate and benzoic acid. (c) Ratio of different products converted from the starting material EGDB.

Hydrolysis of EGDB catalyzed by HiC and HiC@NU-1000 was monitored by the formation of benzoic acid via ^1^H NMR after 48 h of reaction at 80 °C. Building upon existing HiC catalysis under aqueous conditions (pH 8.5 with 10% glycerol, 10 μM HiC) at 80 °C,^30^ we adjusted the aqueous phase to be 100 mM with 10% glycerol at pH 7.5 to minimize the adverse effect of high pH and salt concentration on NU-1000. Similar to problems faced with PET hydrolysis, EGDB catalysis is also limited by its low solubility in aqueous solutions. At 80 °C, the solubility of EGDB was determined to be ∼1.5 mg/mL in 100 mM Tris buffer with 10% glycerol, but a 2-fold increase in solubility was observed in a 50:50 (DMSO-d_6_:100 mM Tris buffer) mixture with 10% glycerol at ∼3.4 mg/mL, which is the solvent condition we chose for all future reactions. This water/DMSO mixture was found to be essential for the reaction. No activity of HiC@NU-1000 was found under pure aqueous conditions due to the low solubility and mobility of EGDB (Figure S4). HiC was also found to be inactive under pure organic conditions due to the lack of water required to perform the hydrolysis reaction. Preliminary screening of the reaction with an equal amount of free or encapsulated HiC demonstrates the formation of three products: monohydrolyzed 2-hydroxyethyl benzoate, completely hydrolyzed product benzoic acid, and ethylene glycol, all visible by ^1^H NMR spectroscopy (Figures 3 and S5). Chemical shifts of ethylene glycol dibenzoate, 2-hydroxyethyl benzoate, and benzoic acid were determined by monitoring the chemical shifts of each individual standard under the same NMR solvent conditions and the ratio between benzoic acid and ethylene glycol in the product. The product ratio was determined by the protons in the aromatic region. Mesitylene was used as an internal standard to quantify the amount of starting material and each product and to calculate the mass balance of the reaction (Figure S6). Due to the small error from the complete mass balance from the free HiC catalysis in triplicate, all product distributions are reported with mass balance normalized to 100% for ease of comparison. HiC@NU-1000 catalysis was first found to be missing over 20% of the substance based on mass balance. We quickly realized that the benzoic acid product can coordinate to the open metal site on the zirconium node in NU-1000 through solvent-assisted ligand incorporation (SALI),^31^ resulting in an undercalculation of the product yield. The missing product was found upon digestion of the postcatalysis HiC@NU-1000 material in a weak base (0.1 M NaOD/D_2_O)^32^ (Figure 3C). A higher amount of ethylene glycol dibenzoate starting material was consumed by free HiC at 86%, but HiC@NU-1000 shows a higher benzoic acid conversion at 64% compared with free HiC at 57%. The higher consumption of starting material by the free enzyme is likely due to its high availability without a diffusion limitation from the MOF framework. On the other hand, high spatial proximity between the HiC catalysts and the monohydrolyzed product 2-hydroxyethyl benzoate within HiC@NU-1000 likely results in a high local concentration of the catalyst and the partially hydrolyzed 2-hydroxyl benzoate, leading to a more complete hydrolysis into benzoic acid (Figure 3C).

### Analysis of the Catalytic Contribution in HiC@NU-1000

The size matching between the enzyme and MOF pore can provide an unique stabilizing effect especially against denaturing organic solvents,^18^ and sometimes leading to enhanced activity.^33^ The hexagonal channel of NU-1000 (3.2 nm) and the dimension of HiC (2.5 × 3.0 × 4.4 nm^3^) share comparable dimensions. To explore the contribution of the pore confining effect toward HiC catalysis, HiC was also encapsulated in NU-1003, an analog of NU-1000 sharing the same topology but with a larger pore size (Figures S7 and S8).^34^ Results from comparing HiC@NU-1000 and HiC@NU-1003 show a small improvement of HiC encapsulation with a larger channel size but a slower conversion of the EGDB starting material, especially into the completely hydrolyzed product benzoic acid (Table 1, Figure S9). The hydrodynamic size of substrate ethylene glycol dibenzoate in the reaction solution mixture (50% DMSO and 50% 0.1 M Tris buffer with 10% glycerol) is measured to be 0.6 nm by dynamic light scattering (DLS) (Figure S7b). Since the substrate is smaller than any of the pores in NU-1000 and NU-1003 (Figure S7c), its diffusion can occur throughout the particle in both frameworks without significant impact from the pore size. On the other hand, the decreased channel diameter in NU-1000 has a more significant confinement effect on the HiC enzyme, which only exists in the mesopore. HiC is a type of cutinase, which contains a preformed oxyanion hole that does not require interfacial activation to expose its active site.^35^ The lack of interfacial activation implies less conformational change upon ligand binding, and its catalytic process does not undergo significant conformational change. The faster hydrolysis process found in HiC@NU-1000 compared with HiC@NU-1003 demonstrates a favorable confining effect through size matching between the enzyme and the host, which enhances the HiC catalytic activity.

**Table 1:** Comparison between NU-1000 and NU-1003 toward HiC Encapsulation and Hydreolysis Performance

Encapsulation			Catalytic performance		
	Amount (mg)		Ethylene glycol dibenzoate	Benzoic acid	2-Hydroxyethyl benzoate
HiC@NU-1000	0.252	89%	35%	52%	14%
HiC@NU-1003	0.286	99%	40%	40%	21%

**Figure 4:**
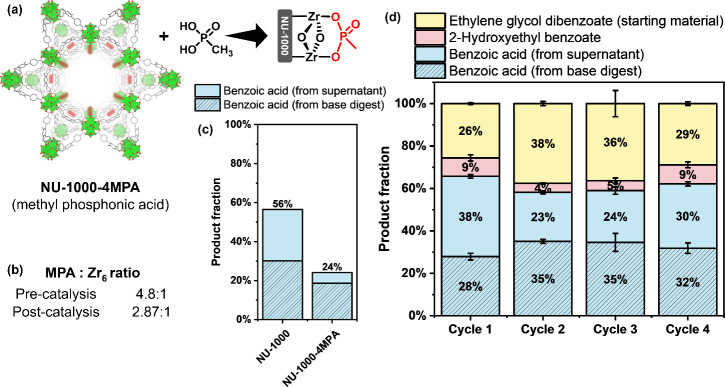
(a) Postsynthetic modification of NU-1000 with methyl phosphonic acid through solvent-assisted ligand incorporation (SALI). (b) Quantification of methyl phosphonic acid pre- and postcatalysis by ^1^H NMR. (c) Yield of benzoic acid from EGDB catalyzed by NU-1000 and NU-1000–4MPA. (d) Yield of various products from EGDB catalyzed by HiC@NU-1000 over four cycles.

The Lewis acidic zirconium node, like the one on NU-1000, is known to catalyze a selection of hydrolysis reaction, notably the phosphonate ester bond in organophosphorus compounds in the presence of base.^36^ More recently, it was also found to hydrolyze peptide bonds.^37^ However, most hydrolysis of carbon-based esters and PET requires prolonged heating over 200 °C.^38^ Despite our condition being much milder than that of reported PET hydrolysis, we still performed control experiments NU-1000 without HiC to quantify the catalytic contribution from the MOF framework. NU-1000 showed up to 56% of benzoic acid conversion (Figures 4C, S10, and S11). To inhibit catalysis on the zirconium node, we capped all open-metal sites with methylphosphonic acid (MPA) through SALI^39^ at 60 °C, and ^1^H NMR confirmed the installation of four ligands per node (Figures 4b and S12). We observed a reduced amount of benzoic acid production from 56% with NU-1000 to 39% with NU-1000–4MPA (Figures 4, S10, and 11). Since SALI was performed at a lower temperature (60 °C) than the catalysis (80 °C), we suspect the lability of the MPA ligands on NU-1000–4MPA leads to the incomplete inhibition of hydrolysis catalysis. A lower MPA/node ratio at 2.5 MPA/node from the postcatalysis NU-1000–4MPA confirmed our hypothesis (Figures 4b and S12). Although the activity contribution in HiC@NU-1000 is lower than that of free HiC, HiC@NU-1000 shows a more complete hydrolysis to benzoic acid, and the heterogeneous HiC@NU-1000 has the potential to be recycled for multiple rounds of catalysis.

### HiC@NU-1000 Recyclability and Its Activity toward PET

In addition to the improved stability and selectivity, protein encapsulation facilitated heterogenization of the biocatalyst, extending its utility. To assess the recyclability of HiC@NU-1000, four sets of HiC@NU-1000 (each containing 0.016 μmol of enzyme) and NU-1000 samples were prepared to monitor their successive rounds of catalytic performance (Round 1, Round 2, Round 3, and Round 4). Following each 48 h catalytic cycle, all HiC@NU-1000 and NU-1000 samples were retrieved via centrifugation and rinsed with 1 mL of water. One set of the MOF and composite samples was subjected to digestion for product determination, while the remainder was transferred to a new experimental vial with fresh substrate for the subsequent catalytic round. The ^1^H NMR spectra of the postcatalysis supernatant and the MOF solution after digestion were analyzed after each cycle, and the conversion percentage of the starting material to benzoic acid was quantified by comparing with a mesitylene standard (Figures S13 and 14). The findings indicated that HiC@NU-1000 maintained catalytic activity across four cycles, with the hydrolysis of the ethylene glycol dibenzoate starting material ranging between 55 and 74% in each round (Figure 4d).

After recycling experiments, we also examined the structural integrity of the resultant materials. The composite particles retained similar morphology and crystallinity (Figure S16) to as-synthesized NU-1000. Furthermore, a leaching assay was conducted to determine whether HiC had escaped from the pores during the catalysis experiments. A Bradford assay was conducted on the postcatalysis solution after each round of catalysis, and minimal leaching was determined (less than 0.2% per round) (Table S1).

**Figure 5:**
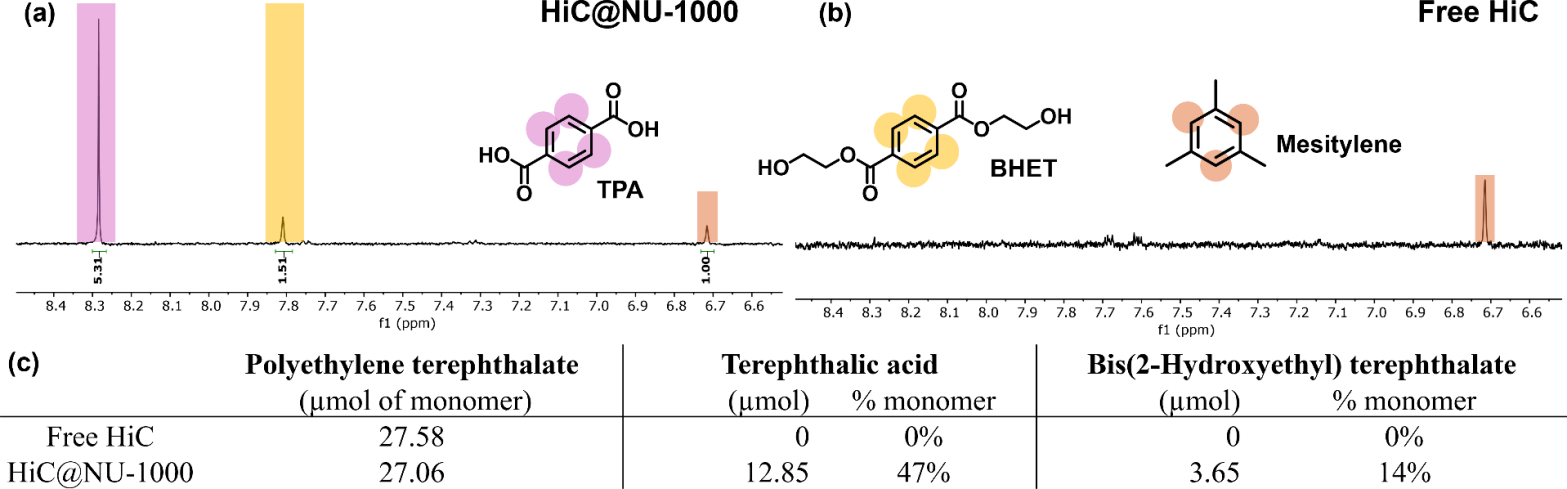
Hydrolysis of polyethylene terephthalate (PET) with HiC@NU-1000 (a) and free HiC (b), with HiC@NU-1000 demonstrating product terephthalic acid (TPA) and bis(2-hydroxyethyl) terephthalate (BHET) but no activity with free HiC. Product was quantified with an internal standard mesitylene and tabulated in (c). Additional TPA and BHET products were also found encapsulated in the NU-1000 pore observed through base digestion of the MOF (Figure S15), which was neglected during the calculation of the yield due to their small amount and the overlapping integrals.

Lastly, we tested free HiC and HiC@NU-1000 for its hydrolysis on PET powder (semicrystalline, 300 μm) under the same hydrolysis conditions on ethylene glycol dibenzoate. Due to the low solubility of PET, the mass balance of the reaction could not be calculated. However, we were able to quantify the product using an internal standard, mesitylene. HiC@NU-1000 was found to be active, converting 47% of the monomer into terephthalic acid and 14% into bis(2-hydroxyethyl) terephthalate^40^ after 48 h at 80 °C while free HiC was found to be not active (Figure 5). The significantly enhanced activity of HiC@NU-1000 can be attributed to both the enhanced stability and availability of the isolated HiC upon encapsulation and the synergic effect from the HiC and Zr_6_ node.

## Conclusion

In this study, we showcased the encapsulation of Humicola insolens Cutinase (HiC), a protein extensively studied for its enzymatic PET depolymerization, to increase its catalytic performance and recyclability in an organic/aqueous solvent mixture. Our findings revealed that the zirconium node in NU-1000 synergistically bolstered the hydrolysis of ethylene glycol dibenzoate (EGDB), while the proximity between the substrate and the HiC catalyst within the NU-1000 framework facilitated a more thorough hydrolysis of the ethylene glycol dibenzoate precursor to benzoic acid. Through recyclability assessments, we observed that HiC@NU-1000 maintained its activity over at least four catalytic cycles with minimal activity loss upon composite recovery. Lastly, we found that HiC@NU-1000 was also active toward the hydrolysis of PET. MOF-encapsulated enzymes hold promise for reducing industrial expenditures through heightened enzyme stability and activity and concurrently enhancing product yields. Our exploration of enzyme encapsulation raises intriguing prospects for future investigations in the field, particularly concerning the potential utilization of the HiC@MOF composite in industrial PET upcycling or analogous enzyme@MOF systems for other pertinent biocatalytic processes in industry.

## Supporting Information Available

Experimental section, which includes materials synthesis, catalysis procedure, characterization (nitrogen sorption isotherms, PXRD, NMR, SEM, HAADF-STEM-EDS, Bradford assay calibration, etc.) (PDF)
