# Supplementary material for: Recyclable Enzymatic Hydrolysis with Metal–Organic Framework Stabilized Humicola insolens Cutinase (HiC) for Potential PET Upcycling
Source: Chem Bio Eng. 2024 Aug 30;1(9):798–804. doi: 10.1021/cbe.4c00101 (PMC11792908; doi:10.1021/cbe.4c00101)
Supplement: Supplementary file 1 — be4c00101_si_001.pdf [file be4c00101_si_001.pdf]

Supplemental Information

Recyclable Enzymatic Hydrolysis with Metal–Organic Framework Stabilized Humicola insolens Cutinase (HiC) for Potential PET Upcycling

Audrianna Wu,[a,†] Fanrui Sha,[a,†] Shengyi Su,[a] Omar K. Farha*,[a, b]

[a] *International Institute for Nanotechnology and Department of Chemistry, Northwestern University, 2145 Sheridan Road, Evanston, Illinois 60208, United States*

[b] *Department of Chemical and Biological Engineering, Northwestern University, 2145 Sheridan Road, Evanston, Illinois 60208, United States*

[†]Authors contributed equally to this paper

*Corresponding author: o-farha@northwestern.edu

KEYWORDS: Protein encapsulation; biocatalysis; metal–organic frameworks (MOFs); polyethylene terephthalate (PET) recycling; chemical upcycling

Contents

Materials S2

Experimental S3

Figure S1. Structural characterization for as-synthesized NU-1000 .... S8

Figure S2. Optimization of HiC@NU-1000 encapsulation, ... S8

Figure S3. Calibration curves of Bradford assay for HiC S9

Figure S4. Hydrolysis of EGDB with free HiC and HiC@NU-1000 in pure aqueous media (100 mM pH 7.5 Tris with 10% glycerol) at 80 °C for 48 hours S9

Figure S5. All hydrolysis products catalyzed by HiC@NU-1000 identified by ^{1}H NMR spectrum from the reaction supernatant. S10

Figure S6. Product formation from free HiC catalysis based on triplicated experiments S10

Figure S7. Comparison between the dimensions of HiC, ethylene glycol benzoate, and the pore size of NU-1000 and NU-1003 .... S11

Figure S8. (a) PXRD and (b) SEM of as synthesized NU-1003 at 1 μm. S11

Figure S9. The comparison between the encapsulation and hydrolysis of HiC@NU-1000 and HiC@NU-1003 .... S12

Figure S10. Product determination from hydrolysis by NU-1000, NU-1000-4MPA, and HiC@NU-1000-4MPA S12

Figure S11. Product distribution from NU-1000 and NU-1000-4MPA control experiments S13

Figure S12. 1H NMR spectrum of digested NU-1000-4MPA before and after catalysis S13

Figure S13. Product determination from four cycles of HiC@NU-1000 catalysis via ^{1}H NMR spectrum of reaction supernatant S14

Figure S14. Product determination from four cycles of HiC@NU-1000 catalysis via ^{1}H NMR spectrum of the base digested HiC@NU-1000 .... S14

Figure S15. Structural characterization for HiC@NU-1000 post-catalysis S15

Figure S16. Comparison between base digested HiC@NU-1000 containing TPA and BHET and clean H_4TBAPy linker S15

Table S1. Amount of HiC leached from each round of HiC@NU-1000 catalysis S16

Materials

All chemicals were purchased from the suppliers and used as received. In all experiments, water was Milli-Q (Millipore). Brilliant Blue G-250, zirconyl chloride octahydrate (≥ 98%), benzoic acid (≥ 99.5%), trifluoroacetic acid (TFA) (≥ 99%), ethylene glycol dibenzoate, and methyl phosphonic acid were purchased from Sigma-Aldrich. Glycerol, 1 M Tris Buffer (pH 7.5), hydrochloric acid (37%), *N,N*-dimethylformamide (DMF) (99.9%), acetone (99.8%), 85% phosphoric acid were purchased from Fisher Scientific. Humicola insolens Cutinase (HiC), also known as Novozym® 51032 was purchased from STREM Chemicals Inc. Polyethylene terephthalate (PET) powder (semicrystalline, 300 µm) was purchased from goodfellow advanced materials (UOM code: 862-403-03). Bradford assay solution[1] and H_4TBAPy[2] were prepared according to reported procedure.

Experimental

Instrumentation

The crystallinity of NU-1000 and HiC@NU-1000 was verified with powder X-ray diffraction (PXRD) collected on a STOE-STADI-P powder diffractometer with CuKa1 radiation (λ = 1.54056 Å) and compared to simulated NU-1000 patterns. ^{1}H NMR spectra of samples were obtained using a Bruker Avance III 500 MHz spectrometer at the Northwestern IMSERC facility. Scanning electron microscopy (SEM) images of both NU-1000 and HiC@NU-1000 were collected on Hitachi SU8030 after 18 nm of Os plasma coating. Transmission electron microscopy (TEM) samples were prepared by pipetting ~4 µL of sample (1 mg/mL) dispersed in methanol onto a 200-mesh copper TEM grids with lacey carbon support layer. Transmission electron microscopy (TEM) imaging was performed using a JEOL ARM200CF Aberration-Corrected TEM (JEOL, Ltd., Tokyo, Japan) operated at 200 kV and emission current of 15 µA, equipped with a Gatan OneView CMOS camera, located at Northwestern University's EPIC/NUANCE facility. Micrographs were recorded using Gatan Digital Micrograph image acquisition. High-angle annular dark-field scanning transmission electron microscopy (HAADF-STEM) imaging and energy dispersive X-ray spectroscopy (EDS) elemental mapping were performed using the same JEOL ARM200CF Aberration-Corrected TEM. X-ray signals were collected using a Dual SDD EDS detector and processed using the Pathfinder X-ray microanalysis software. The porosity of NU-1000 was measured using isothermal N_2 adsorption at 77 K on a Micromeritics Tristar II 3020 instrument. The pore size distributions were calculated using a DFT model with slit geometry. Prior to N_2 adsorption measurements, a 50-60 mg sample of NU-1000 was activated at 120°C under vacuum on a Smart VacPrep instrument from Micromeritics. Protein assays were analyzed with BioTek Synergy Neo2 multimode microplate reader. Dynamic light scattering measurement was performed on Malvern Zetasizer Nano in a solution containing 50% DMSO and 50% 100 mM Tris buffer with 10% glycerol.

Synthesis of NU-1000

NU-1000 [$Zr_6(\mu_3\text{-}O)_4(\mu_3\text{- }OH)_4(H_2O)_4(OH)_4(TBAPy)_2$] and the corresponding H_4TBAPy linker 4,4',4'',4'''-(pyrene-1,3,6,8-tetrayl)tetrabenzoic acid were synthesized according to published procedures. [1,2] Specifically, $ZrOCl_2\cdot 8H_2O$ (1.45 g, 4.52 mmol) and benzoic acid (30 g, 246 mmol)

were dissolved in 90 mL dimethylformamide (DMF) in a 100-mL glass bottle and sonicated for 10 min. The clear solution was incubated in an oven at 100°C for 1 h. H_4TBAPy linker (0.6 g, 0.879 mmol) was dissolved in 30 mL DMF, sonicated for 10 min, and heated to 100°C for 1 h. After cooling to room temperature, the H_4TBAPy solution and trifluoroacetic acid (0.6 mL, 7.83 mmol) were combined and added to the pre-made Zr node containing solution and sonicated for 10 min. The yellow suspension was placed in a pre-heated oven at 120°C for 18 h. After cooling to room temperature, the yellow powder was collected into four 50-mL centrifuge tubes by centrifugation (5 min, 7500 rpm) and washed with DMF three times (~100 mL each, soaked ~1 h between washes). Then the yellow powder was suspended in 195 mL DMF in a 200-mL glass bottle, and 7.5 mL of 8 M aqueous HCl was added. This mixture was heated in an oven at 100°C for 18 h. After cooling to room temperature, the powder was isolated by centrifugation and washed with DMF three times (~200 mL each, soaked ~1 h between washes) and acetone three times (~200 mL each, soaked ~1 h between washes) and soaked in acetone for an additional 16 h. NU-1000 crystals were collected by centrifugation and dried in a vacuum oven at 80°C overnight, and then activated using a Micromeritics Smart VacPrep instrument at 120°C under vacuum prior to characterization and experiments.

Synthesis of NU-1003

Linker for NU-1003 H_4TNAPy was synthesized cording to published procedures.[3] For the synthesis of 1 µm NU-1003, solution A was prepared with 200 mg $ZrOCl_2 \cdot 8H_2O$ dissolved in 50 mL DMF with 1.1 mL TFA, and solution B was prepared with 48 mg H_4TNAPy linker dissolved in 50 mL DMF. In a 10-20 mL microwave vial, 10 mL A and 10 mL B were combined and ran immediately on the microwave at 160 °C for 15 minutes. Yellow crystallites were isolated by centrifuging and washed with DMF 3 times (20 mL each, soaked ~1 h between washes), acetone 3 times (20 mL each, soaked ~1 h between washes), and exchanged into water right before the encapsulation experiment.

HiC encapsulation optimization

To determine the optimal encapsulation condition, 0 mM (water), 100 mM, 200 mM, and 500 mM pH 7.5 Tris buffer with HiC (6.25 µL, 105 µg, density 16.8 mg/mL, 0.006 µmol) and 500 mM, 600 mM, 700 mM, 800 mM, 900 mM, and 1000 mM Tris buffer with HiC (31.25 µL, 525 µg, 0.033 µmol) were investigated for their encapsulation potential. Specifically, 5 mg of NU-1000 was added to 1 mL of buffer solution with appropriate concentration in 1.5 mL Eppendorf tubes and sonicated for 5 min. HiC was added to each tube. Each sample was left on a rotating shaker at room temperature. After 24 h, the HiC@NU-1000 samples were subjected to centrifugation, and all of the supernatant was taken off. A 20 µL aliquot of the supernatant was diluted with 80 µL of water for plating. The composite was then washed with 1 mL of water, vortexed, and a second sample was isolated by centrifugation. The supernatant was subjected to centrifugation again to eliminate MOF particles.

To determine the composite's enzyme loading, the residual HiC left in the supernatant was analyzed using a Bradford Assay on a 96-well plate. A standard calibration curve was prepared between 0-0.00625M HiC in 500 mM Tris buffer. On a 96-well plate, 180 µL of the prepared dye

and 20 µL of each standard or supernatant were added to each well. Absorbance at 595 nm was measured and the amount of residual HiC left in the supernatant was calculated from the standard curve. All analyses were performed in triplicate.

The Bradford dye was prepared according to published procedures.[3] Coomassie Brilliant Blue G-250 (20 mg) was dissolved in 10 mL of 95% ethanol. 20 mL of 85% phosphoric acid was added to this solution. The resulting solution was diluted to a final volume of 200 mL and filtered with a Buchner funnel. The solution was separated into four 50-mL centrifuge tubes, wrapped in aluminum foil to block light, and stored at 4°C.

A final experiment was repeated with 1 ml of 500 mM Tris buffer with 10% glycerol to determine optimal encapsulation conditions.

For the comparison between NU-1000 and NU-1003, encapsulation was performed and evaluated under the optimized condition (5 mg NU-1000 or NU-1003), 17 µL HiC in 1 mL 300 mM Tris buffer with 10 % glycerol for 24 hours.

NU-1000-4MPA preparation

Solvent-assisted ligand incorporation (SALI) was prepared according to published procedures.[4] NU-1000 (45 mg, 0.021 mmol) was added to a 4-dram vial. Subsequently 0.027 M methylphosphonic acid (9.79 mg, 0.102 mmol) in 3.78 mL of DMF. The vial was sealed and heated at 60 °C for 18-24 h with occasional swirling. The solution was transferred to a 10-mL centrifuge tube and subjected to centrifugation at 7000 rpm for 5 min. The supernatant was decanted, the MOF was washed in DMF (5 x 10 mL), acetone (5 x 10 mL, and DCM (3 x 10 mL). The MOF sample was then dried in an 80°C vacuum oven overnight. A small sample of the MOF was then digested with 0.1M NaOD solution, sonicated for 5-10 min, diluted with D_2O, and sonicated for another 5-10 min. The base digest sample was transferred to a ^1H NMR tube via Pasteur pipet and a ^1H NMR spectrum was taken, which shows 4.42 equivalents of methylphosphonic acid/NU-1000 node.

Free HiC, HiC@NU-1000, HiC@NU-1003, and HiC@NU1000-4MPA hydrolysis activity analysis (including NU-1000 and NU-1000-4MPA control)

To evaluate the catalytic activity of free HiC, HiC@NU-1000, HiC@NU-1003, HiC@NU-1000-4MPA, and the contribution from the empty framework (NU-1000 and NU-1000-4MPA), 5.55 mg of HiC@NU-1000 containing 17 µL (286 µg, 0.016 µmol) of HiC, 5.00 mg of HiC@NU-1003 containing 17 µL (286 µg, 0.016 µmol) of HiC, and 5.12 mg of HiC@NU-1000-4MPA containing 17 µL (286 µg, 0.016 µmol) of HiC composite were prepared according to the optimal encapsulation conditions determined above. This HiC@NU-1000 composite was compared to a 17 µL free enzyme control, 5.58 mg of NU-1000, 5.00 mg of HiC@NU-1003, and 5.40 mg of NU-1000-4MPA control. Each catalyst was placed in a 2-mL solution of a 50:50 (d'DMSO:100 mM Tris Buffer) mixture with 10% glycerol in a 1-dram vial. ~ 6 mg of EGDB (specifically, 6.20, 5.90, 6.03, 6.13, 5.40 mg, and 6.15 mg) were added to each vial, respectively. The solutions were sonicated for 3-5 min and placed in an 80°C oven for 48 h. After 48 h, a 0.5 mL sample was taken from each vial and placed in Eppendorf tubes. The samples were subjected to centrifugation to

separate the supernatant from HiC@NU-1000 or NU-1000 in the sample. 10 μL of internal standard solution containing methylene was added to each tube. The solutions were vortexed and then transferred to NMR tubes via Pasteur pipet. A ^{1}H NMR spectrum was taken of each sample. The remaining MOF from each sample was placed in a vacuum oven overnight. A small sample of the dried composite or MOF was then digested with 0.1M NaOD solution, sonicated for 5-10 min, diluted with D_2O, and sonicated for another 5-10 min. The base digest sample was transferred to a ^{1}H NMR tube via Pasteur pipet and a ^{1}H NMR spectrum was taken. Error bars on the activity of free HiC and HiC@NU-1000 hydrolysis were obtained through three independent trials.

HiC@NU-1000 and NU-1000 recyclability and leaching analysis

To test HiC@NU-1000 recyclability, 17 μL (286 μg, 0.016 μmol) of HiC were encapsulated in ~5 mg of NU-1000 (specifically, 5.04, 5.04, 5.07, and 5.09 mg) according to the optimal encapsulation conditions determined above. The samples were labeled as samples #1-4 for Rounds 1, 2, 3, and 4, respectively. Each HiC@NU-1000 composite was paired with ~5 mg of NU-1000 (specifically, 5.04, 5.03, 5.02, and 5.00 mg) which would complete the same number of catalytic rounds as their counterparts.

For the first round of catalysis, 2 mL of a 50:50 mixture of d'DMSO:100 mM Tris Buffer with 10% glycerol was prepared and added to four 1-dram vials. ~5.90 mg of EGDB (specifically, 5.90, 5.96, 5.93, and 5.89 mg) was added to each vial, labeled #1-4. The HiC@NU-1000 composite corresponding to each number was added to the solution. The corresponding NU-1000 samples were added to 2 mL of a 50:50 mixture of d'DMSO:100 mM Tris Buffer with 10% glycerol, with 5.85, 5.96, 5.91, and 5.89 mg of EGDB, respectively. Additionally, a free enzyme control (17 μL, 286 μg, 0.016 μmol) was added to a separate vial with 2 ml of a 50:50 mixture of d'DMSO:100 mM Tris Buffer with 10% glycerol and 5.92 mg of EGDB. All samples were sonicated for 5-10 min, and the vials were placed in an 80°C oven for 48 h.

After 48 h, the vials were removed from the oven, and three 0.5 mL aliquots were taken from both vials labeled as #1 (for 1 round of catalysis only) and placed in separate Eppendorf tubes. The solutions were subjected to centrifugation and the supernatant was taken off and placed in new Eppendorf tubes. 10 μL of internal standard solution containing methylene was added to each tube. The solutions were vortexed and then transferred to NMR tubes via Pasteur pipet. A ^{1}H NMR spectrum was taken of each sample.

The remaining HiC@NU-1000 composite in the Eppendorf tube was placed in a vacuum oven overnight. Three separate samples of the dried composite were digested with 0.1M NaOD solution, sonicated, and then diluted with D_2O. The base digest samples were transferred to ^{1}H NMR tubes via Pasteur pipet and a ^{1}H NMR spectrum was taken of each sample. The remaining reaction solution from each vial was placed in a second Eppendorf tube and subjected to centrifugation. The supernatant was removed and placed in a separate Eppendorf tube and stored in the fridge for a leaching assay.

For the paired samples for Round 2, 3, and 4 analyses, the HiC@NU-1000 composite and NU-1000 samples were retrieved via centrifugation after the previous round of catalysis and washed with 1 mL of water. Similar procedures were carried out to analyze catalysis and enzyme leaching

from the supernatant solution and the HiC@NU-1000 particles and set up for the next round of catalysis at 80 °C. For the second round of catalysis, EGDB was massed to be 5.92, 5.95, and 5.90 mg for vials labeled #2-4, respectively. For the third round of catalysis, EGDB 5.93 and 5.93 mg of EGDB were added to HiC@NU-1000 vials labeled #3-4, and 5.89 mg of EGDB was added to vial #4 for the fourth round of catalysis.

After the catalytic portion of the experiment was completed, the amount of enzyme that had leaked out from the HiC@NU-1000 composite after each subsequent round of catalysis was analyzed using a Bradford Assay on a 96-well plate with the remaining supernatant stored in the fridge. A standard calibration curve was prepared between 0-0.00625M HiC in a 50:50 mixture of d'DMSO:100 mM Tris buffer with 10% glycerol. On a 96-well plate, 180 μL of the prepared dye and 20 μL of each standard or supernatant were added to each well. Absorbance at 595 nm was measured and the amount of residual HiC left in the supernatant was calculated from the standard curve. All analysis was performed in triplicate to obtain error bars.

Hydrolysis of PET powder with HiC@NU-1000

HiC@NU-1000 was prepared following the optimized encapsulation procedure, where 17 μL of HiC solution was dissolved in 1 mL of 500 mM Tris buffer at pH 7.5 containing 10% glycerol. To the solution, 5 mg of NU-1000 was dispersed by 5 minutes of sonication, then put on a rotator for 24 hours. Bradford assay after 24 hours revealed about 88% of HiC was removed from the supernatant and encapsulated in NU-1000 (equivalent of 15 μL of HiC solution). Hydrolysis experiment with HiC@NU-1000 was set up in 1-dram vial, with 5 mg of HiC@NU-1000 added to a 2 mL solution containing 1 mL DMSO-d_6 and 1 mL of 100 mM Tris buffer solution containing 10% glycerol and 5.2 mg of PET. To the solution, 15 μL (16.4 mg) of 10% mesitylene solution was added (90.4 mg in 1168.2 mg of DMSO-d_6) as internal standard. Free HiC was also set up as a comparison in a 1-dram vial, with 15 μL native HiC dissolved in a 2 mL solution containing 1 mL DMSO-d_6 and 1 mL of 100 mM Tris buffer solution containing 10% glycerol and 5.3 mg of PET. To the solution, 20 μL (22.25 mg) of 10% mesitylene solution was also added (90.4 mg in 1168.2 mg of DMSO-d_6) as an internal standard. Both vials were incubated at 80 °C for 24 hours, and the results were analyzed by ^{1}H NMR.

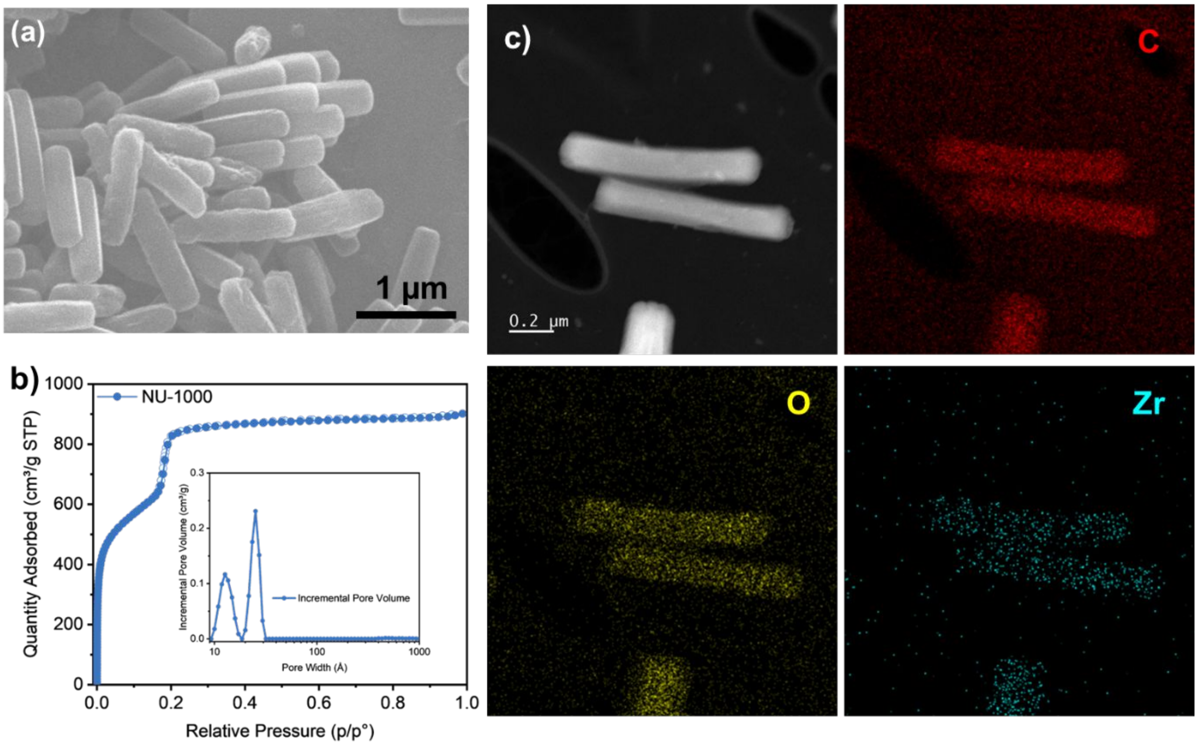

Figure S1. Structural characterization for as-synthesized NU-1000 (a) SEM image of NU-1000, (b) N_2 sorption isotherm of NU-1000 and its DFT-calculated pore size distribution, and (c) HAADF-STEM image and STEM-EDS mapping signal for spatial distribution of carbon, oxygen, and zirconium elements in NU-1000.

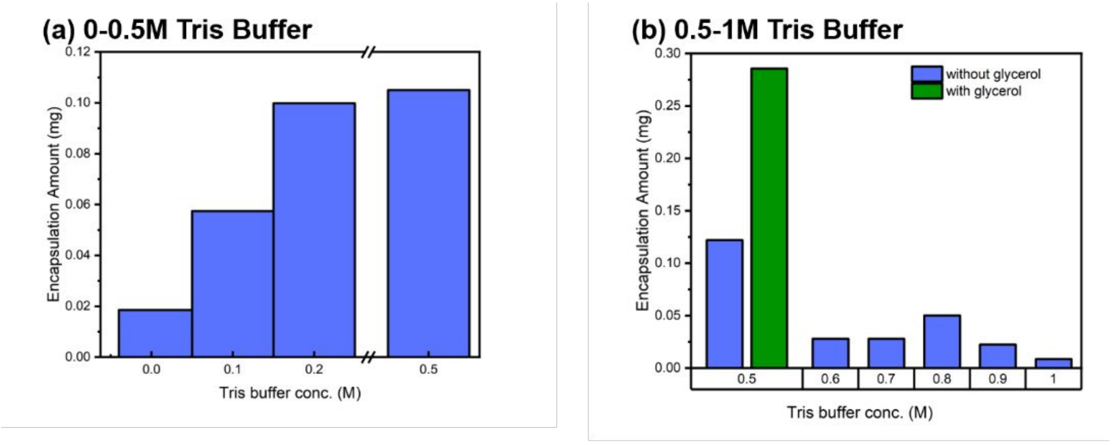

Figure S2. Optimization of HiC@NU-1000 encapsulation by (a) varying the buffer concentration and (b) the addition of 10% glycerol, showing the amount of HiC encapsulated in NU-1000 determined by Bradford Assay in differing buffer concentration.

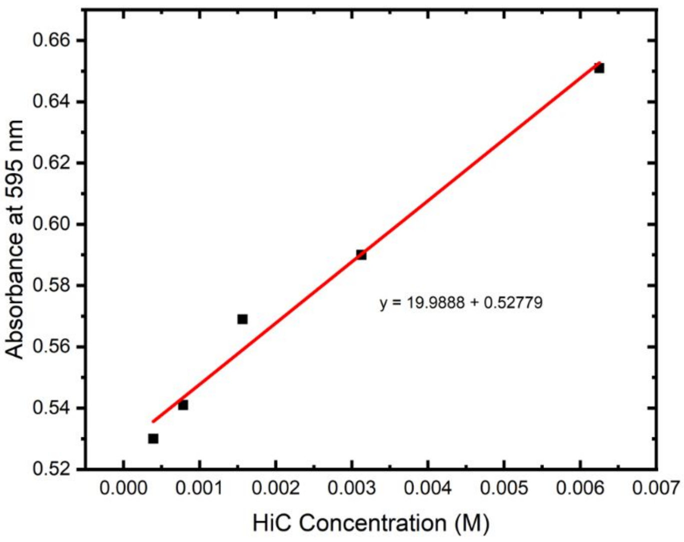

Figure S3. Calibration curves of Bradford assay for HiC encapsulation using serial dilutions of HiC in 500 mM Tris (10% glycerol).

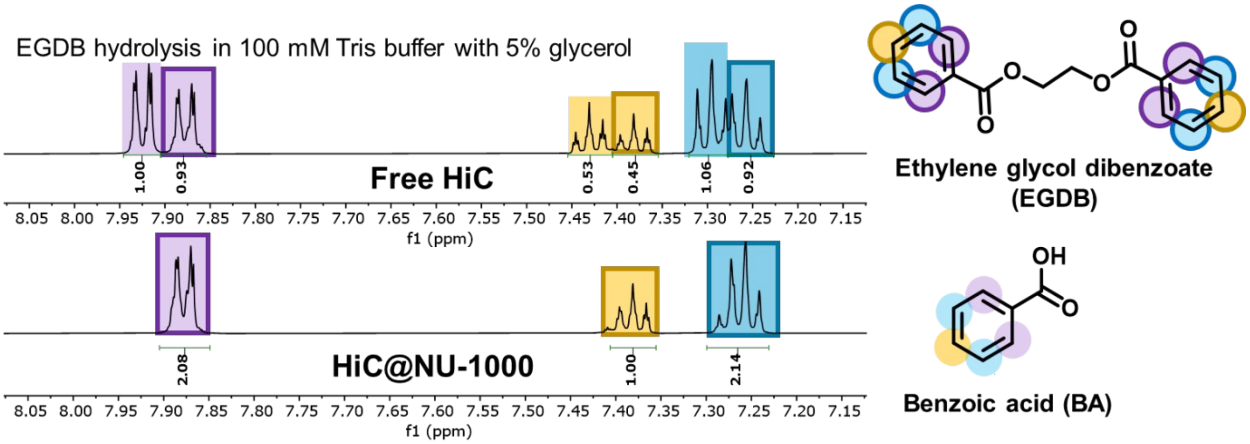

Figure S4. Hydrolysis of EGDB with free HiC and HiC@NU-1000 in pure aqueous media (100 mM pH 7.5 Tris with 10% glycerol) at 80 °C for 48 hours. Free HiC was found to be active but not HiC@NU-1000.

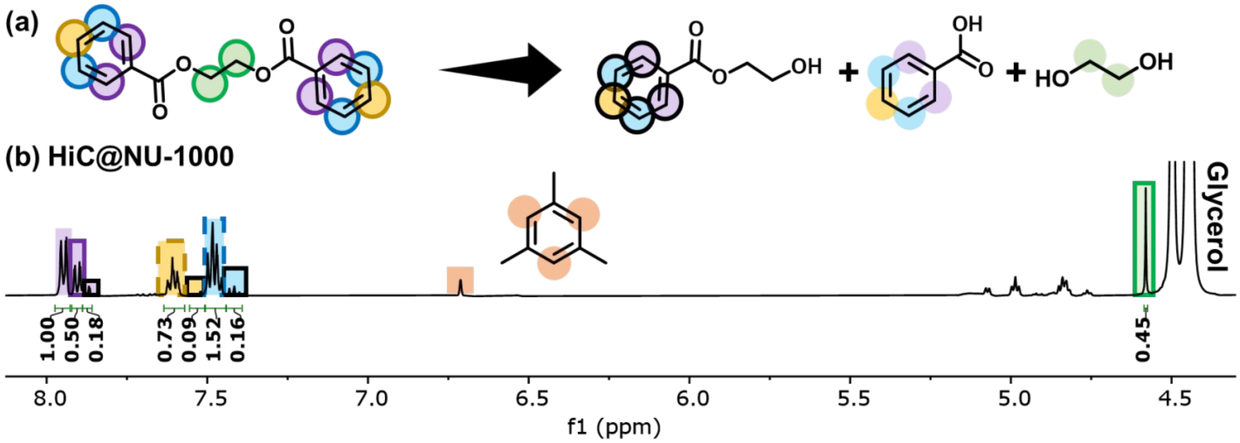

Figure S5. All hydrolysis products catalyzed by HiC@NU-1000 identified by ^{1}H NMR spectrum from the reaction supernatant. The reaction scheme (a) illustrates ethylene glycol dibenzoate starting material and all possible products and (b) their characterization by ^{1}H NMR in (b). Only the aromatic region (between 6.5 to 8.5 ppm) was used for product quantification due to the occasional overlap between ethylene glycol, glycerol, and water.

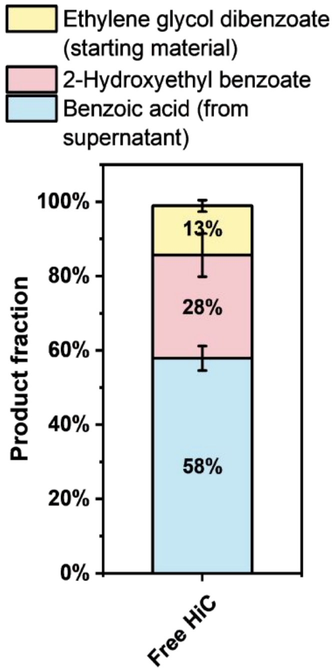

Figure S6. Product formation from free HiC catalysis based on triplicated experiments, demonstrating good mass balance with error bar on the conversion of each product at below 5%.

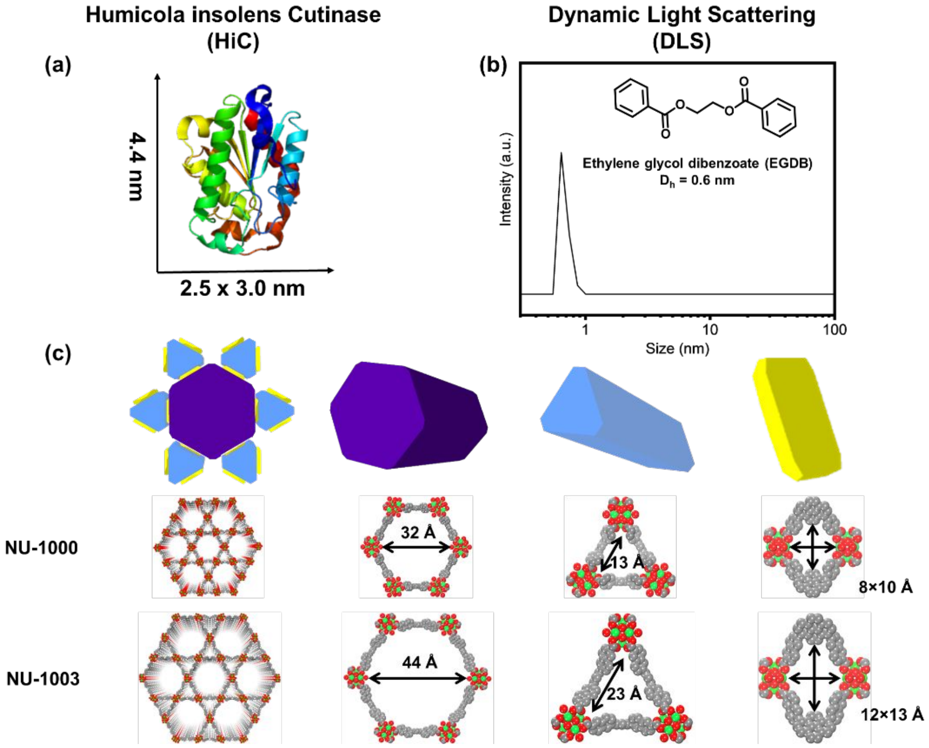

Figure S7. Comparison between the dimensions of the (a) HiC protein based on its crystal structure, (b) hydrodynamic diameter (D_h) of substrate ethylene glycol dibenzoate measured by DLS to be 0.6 nm, and (c) the pore dimensions of NU-1000 and NU-1003. Figure S7(c) adopted with copyright from Elsevier and Copyright Clearance Center.[3]

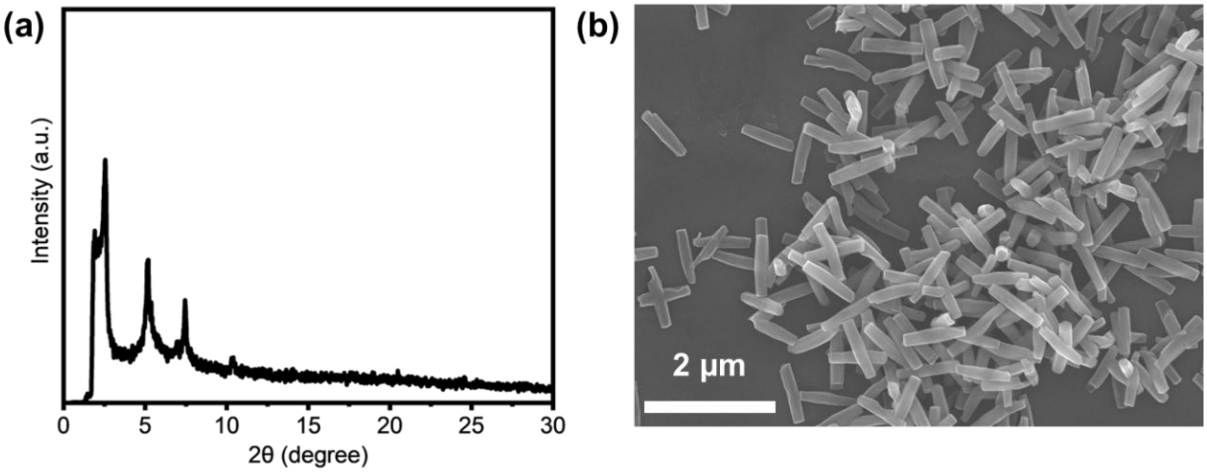

Figure S8. (a) PXRD and (b) SEM of as synthesized NU-1003 at 1 μm.

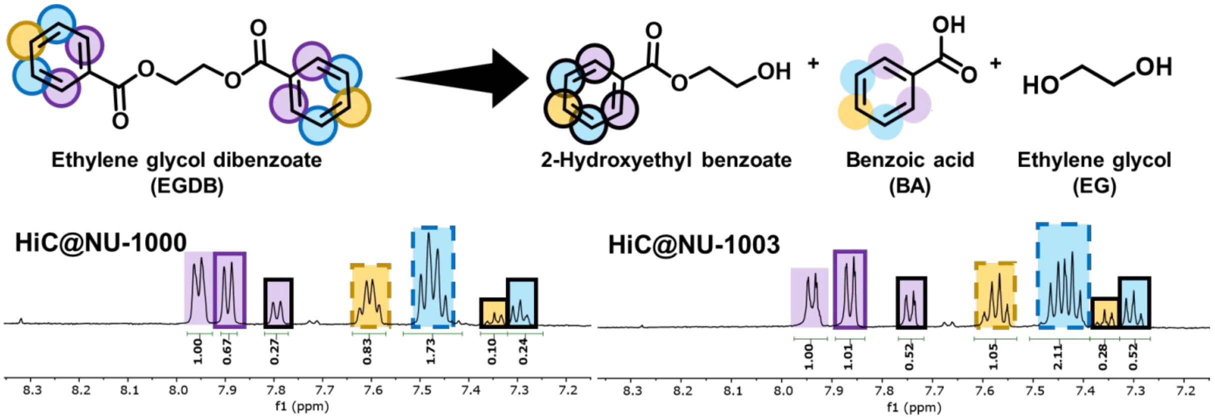

Figure S9. The comparison between the encapsulation and hydrolysis of HiC@NU-1000 and HiC@NU-1003.

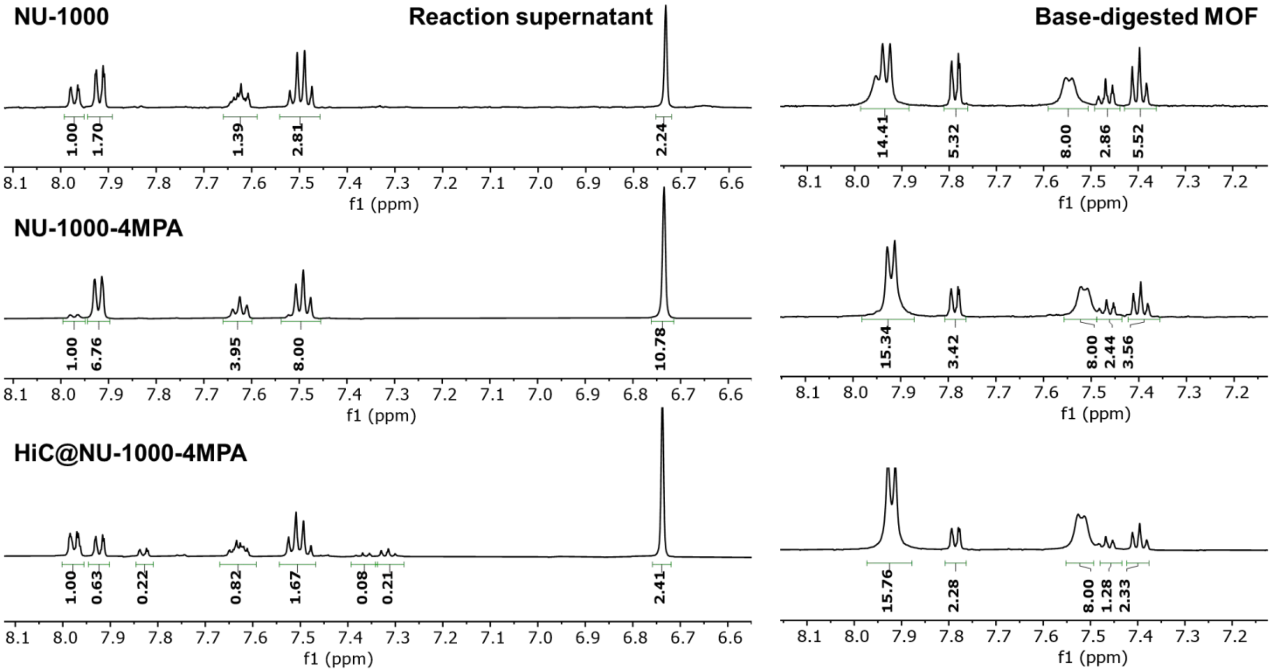

Figure S10. Product determination from hydrolysis by NU-1000, NU-1000-4MPA, and HiC@NU-1000-4MPA via ^{1}H NMR spectrum of reaction supernatant and the base-digested MOF.

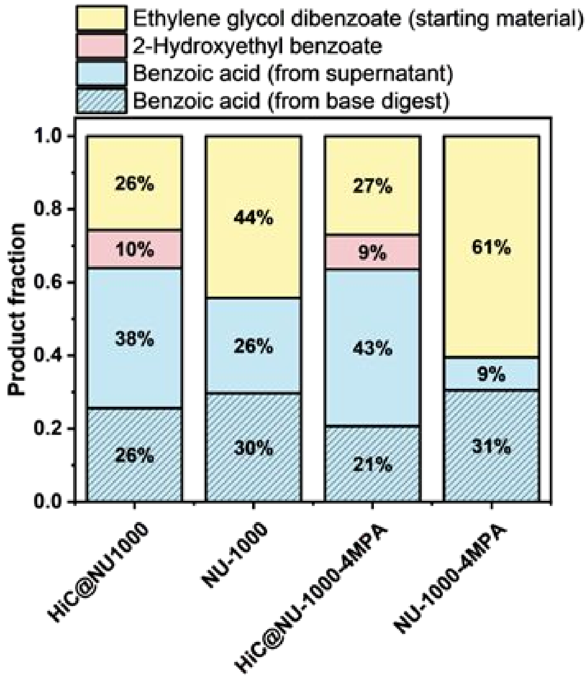

Figure S11. Product distribution from NU-1000 and NU-1000-4MPA control experiments.

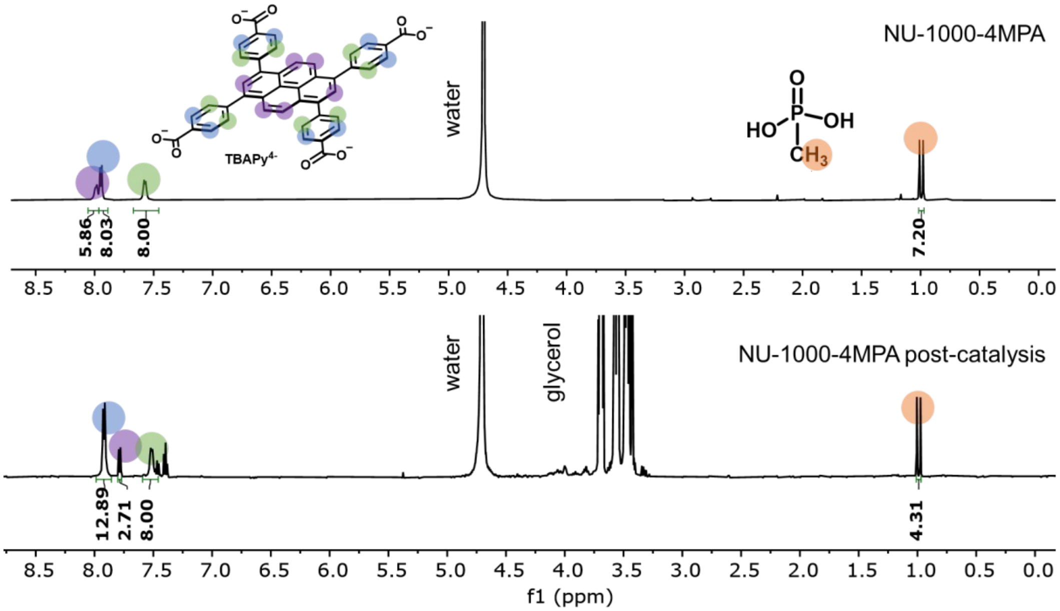

Figure S12. 1H NMR spectrum of digested NU-1000-4MPA before and after catalysis.

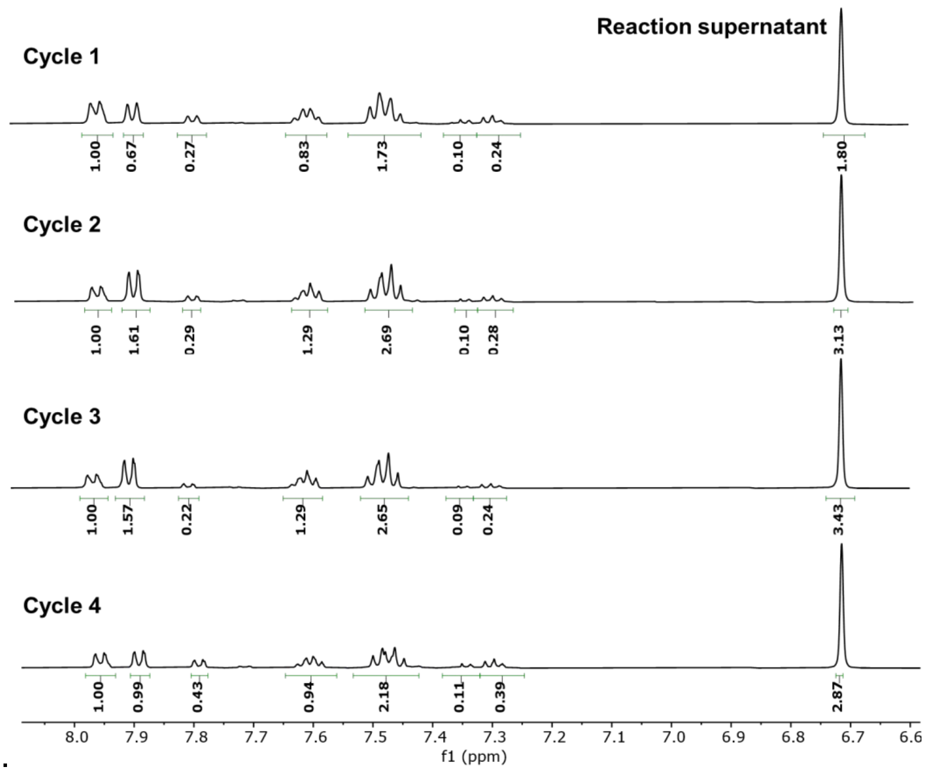

Figure S13. Product determination from four cycles of HiC@NU-1000 catalysis via ^{1}H NMR spectrum of reaction supernatant.

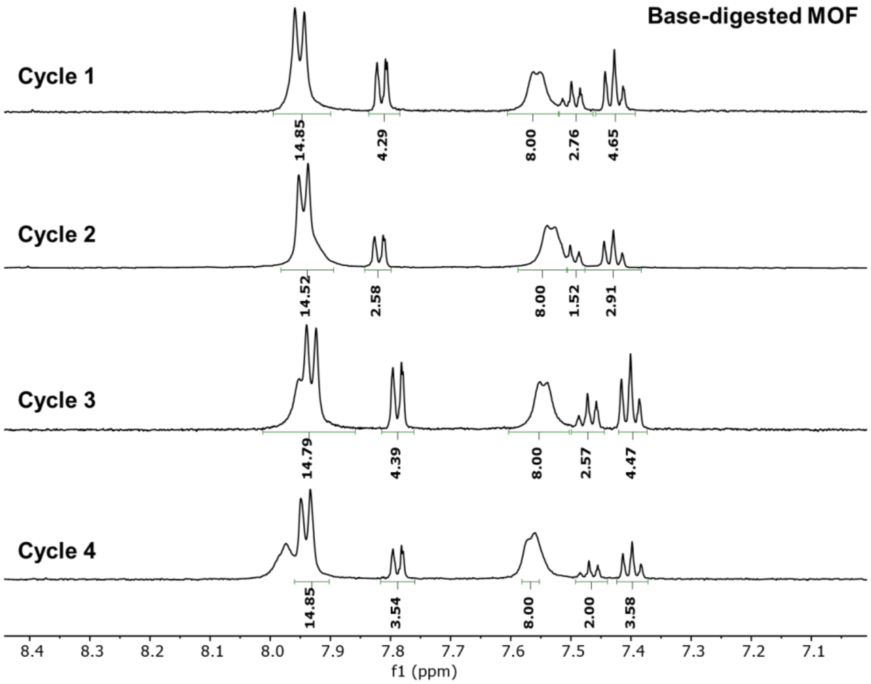

Figure S14. Product determination from four cycles of HiC@NU-1000 catalysis via ^{1}H NMR spectrum of the base digested HiC@NU-1000.

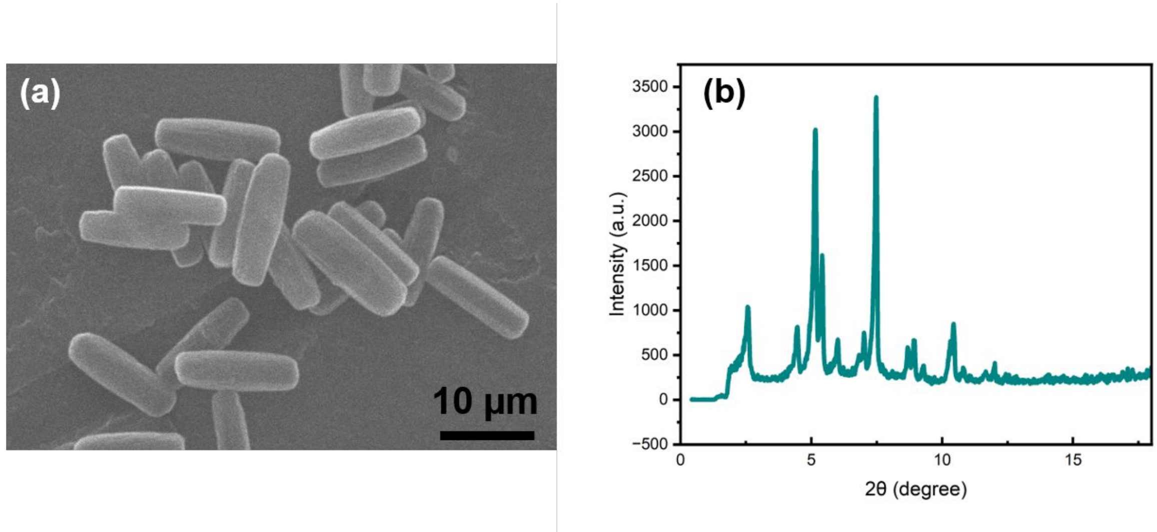

Figure S15. Structural characterization for HiC@NU-1000 post-catalysis (a) SEM image for HiC@NU-1000 post-catalysis (b) PXRD pattern for HiC@NU-1000 post-catalysis.

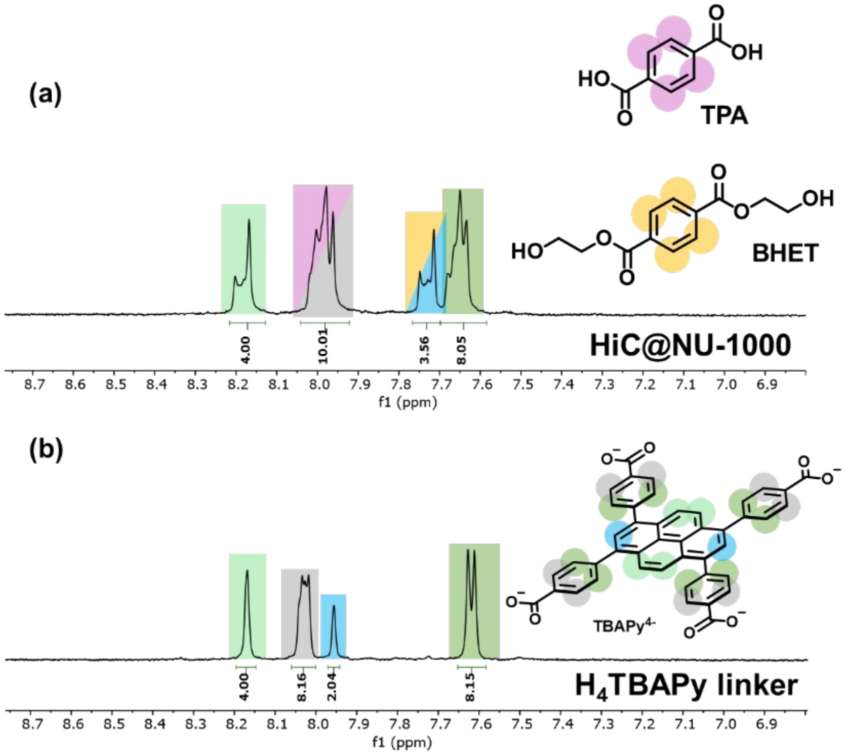

Figure S16. Comparison between (a) base digested HiC@NU-1000 containing TPA and BHET and (b) clean H_4TBAPy linker

Table S1. Amount of HiC leached from each round of HiC@NU-1000 catalysis

Round number	µl of HiC leaked from HiC@NU-1000
1	0.00549
2	0.00726
3	0.00799
4	0.00488

(1) Bradford, M. M. A Rapid and Sensitive Method for the Quantitation of Microgram Quantities of Protein Utilizing the Principle of Protein-Dye Binding. *Anal. Biochem.* **1976**, 72, 248–54.

(2) Wang, T. C.; Vermeulen, A. V.; Kim, I. S.; Martinson, A. B. F.; Stoddart, J. F.; Hupp, J. T.; Farha, O. K. Scalable Synthesis and Post-Modification of a Mesoporous Metal-Organic Framework Called NU-1000. *Nat. Protoc.* **2016**, 11, 149–62.

(3) Li, P.; Chen, Q.; Wang, T. C.; Vermeulen, N. A.; Mehdi, B. L.; Dohnalkova, A.; Browning, N. D.; Shen, D.; Anderson, R.; Gómez-Gualdrón, D. A.; Cetin, F. M., Jagiello, J.; Asiri, A. M., Stoddart, J. F.; Farha, O. K. Hierarchically Engineered Mesoporous Metal–Organic Frameworks toward Cell-free Immobilized Enzyme Systems. *Chem* **2018**, 1022-1034.
